# Ribozyme-Mediated Inhibition of 801-bp Deletion-Mutant *Epidermal Growth Factor Receptor* mRNA Expression in Glioblastoma Multiforme

**DOI:** 10.3390/molecules15074670

**Published:** 2010-06-30

**Authors:** Georg Karpel-Massler, Christian Rainer Wirtz, Marc-Eric Halatsch

**Affiliations:** Department of Neurosurgery, University of Ulm Medical School, Steinhövelstr 9, D-89075 Ulm, Germany; E-Mails: georg.karpel@uniklinik-ulm.de (G.K.-M.); rainer.wirtz@uniklinik-ulm.de (C.R.W.)

**Keywords:** EGFRvIII, ribozymes, retrovirus-mediated DNA transfer

## Abstract

The epidermal growth factor receptor (HER1/EGFR) is known to be disregulated in a large subgroup of glioblastoma multiforme cases. Disregulation of HER1/EGFR is related to malignant transformation and tumor growth in various human cancers, including malignant glioma. One mechanism that may lead to disregulated HER1/EGFR signaling is the intrinsic alteration of the receptor structure due to mutational changes. The most common mutant form of HER1/EGFR, named variant III (EGFRvIII), results from an 801 bp in-frame deletion in the DNA sequence encoding the extracellular ligand-binding domain. Independent of ligand–binding, EGFRvIII is constitutively activated and beyond external control. Since its cellular expression was shown to relate enhanced tumorigenicity, various therapeutic strategies were developed to target EGFRvIII, including monoclonal antibodies, vaccination therapies and small-molecule tyrosine kinase inhibitors. In this review, we focus on ribozyme-mediated inhibition of EGFRvIII messenger RNA expression as a gene therapeutic approach for EGFRvIII-expressing glioblastoma multiforme.

## 1. Introduction

The epidermal growth factor receptor (HER1/EGFR) is a 170 kDa single-chain transmembrane glycoprotein that belongs to the HER family of receptors. It consists of three functional domains, *i.e.*, an extracellular ligand-binding domain, a transmembrane domain and an intracellular tyrosine kinase (TK) domain. Its most common ligands are the epidermal growth factor (EGF) and transforming growth factor-α (TGF-α). Once a ligand binds to the extracellular ligand-binding site of HER1/EGFR, activation of the intracellular TK occurs which triggers downstream signaling via the ras-raf-mitogen-activated protein kinase (MAPK) or the phosphatidylinositol 3-kinase (PI3-K)/Akt pathways. Subsequently, diverse cellular functions are regulated including cellular proliferation and differentiation. HER1/EGFR was found to be disregulated in various human cancers including high-grade glioma. About 50% of glioblastomas, the most malignant and most common type of intrinsic brain tumor in adults, were found to overexpress HER1/EGFR. 

**Figure 1:**
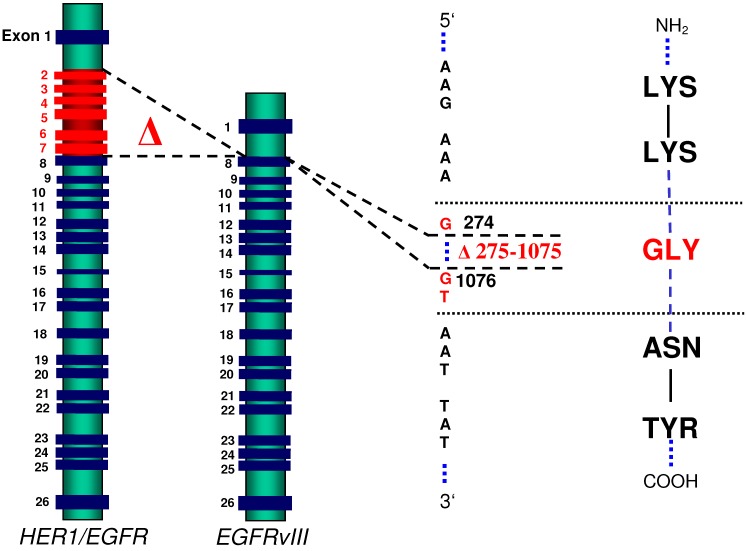
Basic structure of the *wtHER1/EGFR* and the *EGFRvIII* genes, the latter being characterized by a deletion of exons 2-7 comprising 801 basepairs. As a result, a new glycine codon is formed at the fusion junction.

The gene that encodes HER1/EGFR is located on the short arm of chromosome 7 (p11-p13) [1]. It consists of 26 exons and is 110 kb in size. Several mutations of the HER1/EGFR gene have been reported [2]. The most frequent mutant form, arising from rearrangements, is EGFR variant III (EGFRvIII; alternatively termed ΔEGFR), which accounts for approximately 60% of all HER1/EGFR gene aberrations. Elimination of a DNA fragment containing exons 2-7 of the gene results in an in-frame deletion of 801 bp (nucleotide positions 275-1075) of the coding sequence of the extracellular receptor domain and subsequent removal of NH_2_-terminal amino acid residues 6 to 273 from the extracellular domain of the intact wild-type HER1/EGFR (Figure 1) [3,4]. At the fusion junction, a novel glycine residue is created at position 6 in-between the former amino acid residues 5 and 274 [5]. 

EGFRvIII is characterized by constitutive (ligand-independent) activation and defective receptor down-regulation due to low rates of receptor endocytosis. Moreover, expression of EGFRvIII has been shown *in vivo* to be related to increased proliferation, tumor formation and inhibition of apoptosis, affording enhanced tumorigenicity [6,7]. These aspects and the additional fact that EGFRvIII is overexpressed by up to 58% of glioblastomas, but not expressed by normal tissues, renders the mutant receptor an interesting target for the treatment of glioblastoma multiforme. Various therapeutic strategies were developed to target EGFRvIII, including peptide-based vaccination therapy, vector-based targeting of toxins or radiogenic compounds to the receptor or inhibition of the intracellular TK domain by small molecule TK inhibitors [8]. More recently, strategies were explored that interfere with gene expression, including antisense RNA or RNA interference. The fact that as a result of the deletion mutation, the fusion junction of the mutant gene is created directly upstream of a GTA triplet which is subsequently transcribed into a ribozyme target codon (GUA), gave rise to the development of a ribozyme-mediated therapeutic approach [9,10]. In this review, we will focus on the potential role ribozymes may play in the armory against EGFRvIII-expressing glioblastoma multiforme.

## 2. Ribozymes

**Figure 2:**
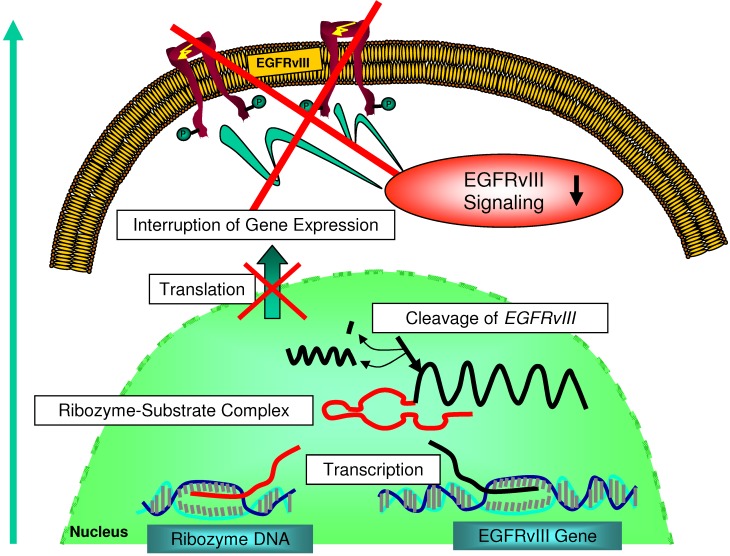
Schematic overview on the mechanism underlying ribozyme-mediated inhibition of *EGFRvIII* gene expression and subsequent downregulation of EGFRvIII-mediated signaling.

The strategy of using RNA molecules for therapeutic purposes is relatively novel. It is based on the realization of the important role RNA plays in the utilization of genetic information but also on the functional diversity of RNAs as molecules that may interact with other RNAs, DNAs, or proteins. Some RNA molecules were shown to form a catalytic center and were termed trans-cleaving ribozymes [11]. Several naturally occuring ribozymes have been identified such as the hammerhead and hairpin types [12,13]. Ribozymes are small RNA molecules that catalytically cleave specific RNA substrates. As a consequence, gene expression and subsequent translation is intercepted (Figure 2). 

The specific properties of a ribozyme are determined by two functional units. The first unit consists of complementary flanking sequences that bind to the substrate RNA by base-pairing interactions and provide specific recognition of the substrate. The second unit is represented by the ribozyme’s catalytic core which mediates site-specific strand scission. Cleavage of the RNA phosphodiester backbone is based on a transesterification reaction resulting in 5’-hydroxyl and 2’-3’-cyclic phosphate termini [13]. 

A search of GenBank revealed that the sequence of the fusion junction of *EGFRvIII* mRNA is unique and missing in the genetic sequence of any known human gene. Thus, highly specific inhibition of *EGFRvIII* mRNA expression might be obtained by ribozymes with complementary flanking sequences designed to recognize the fusion junction.

The best-characterized ribozymes are the hammerhead and hairpin types. The hammerhead, with approximately 40 nucleotides, is the smallest naturally occurring ribozyme [12]. Similar to the hairpin type, the hammerhead represents an essential component of the rolling-circle replication mechanism within virus-like RNAs infecting plants [13]. It is characterized by a “Y-shape” conformation consisting of three helical stems of variable sequence and of 11 highly conserved nucleotides clustered in the center of the three-way junction forming the catalytic core [14]. 

**Figure 3:**
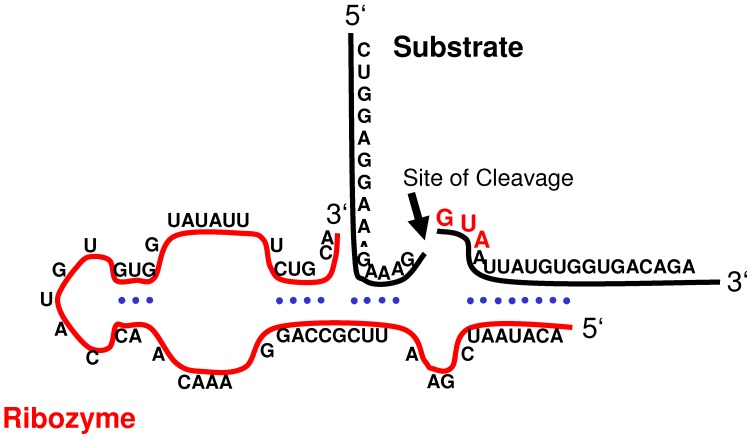
Illustration of the molecular interaction between the sCYMV1-derived hairpin ribozyme and *EGFRvIII* mRNA (substrate). The cleavage site is indicated by an arrow.

Hairpin ribozymes, consisting of approximately 70 nucleotides, are larger than hammerhead ribozymes and possess about the same catalytic efficiency at lower magnesium concentration requirements which may be advantageous for hairpin ribozyme activity *in vivo* [15]. Three different hairpin ribozyme motifs derived from the negative strands of satellite RNAs from tobacco ringspot virus (sTRSV), chicory yellow mottle virus (sCYMV1; Figure 3) and arabis mosaic virus (sArMV), have been described so far [16].

## 3. Ribozyme-mediated Inhibition of *EGFRvIII* mRNA Expression

Most unfortunately, current treatment regimens for glioblastoma multiforme still provide only modest increases in overall survival and are typically not curative [8]. In this situation, research has been spurred to develop other therapeutic strategies including gene therapy. Ribozyme-mediated inhibition of mRNAs that promote tumorigenesis is one such approach.

The question whether a ribozyme specifically targeting EGFRvIII could achieve reversal of the EGFRvIII-associated malignant cellular phenotype was addressed by three experimental studies [9,10,24]. The ribozymes used in these studies were specifically designed to cleave the GUA sequence located directly downstream of the fusion junction in *EGFRvIII* mRNA (Figure 3). The target codon is located within the sequence coding for the receptor’s extracellular domain; considering the ligand-independent activation to be a result of the deletion mutation, this locus may be regarded as essential for autonomous EGFR function. In addition, the mRNA 5’-fragment carrying the translation initiation codon comprises only exon 1; thus it appears reasonable to assume that the cleavage products are non-functional even if translated into polypeptides.

Halatsch *et al.* created three different hairpin ribozymes to specifically target *EGFRvIII* mRNA. Among these three ribozymes, the sCYMV1 motif was identified in a cell-free system to be the most promising candidate molecule against *EGFRvIII*, based on this motif’s cleavage efficacy at physiological pH, temperature, and Mg^2+^ concentration as well as the non-interference with *wtEGFR* [9]. Cleavage was detected at a MgCl_2 _concentration of only 0.1 mM, corresponding to approximately 20% of the intracellular Mg^2+^ concentration. The sCYMV1 hairpin ribozyme motif was then cloned into the retroviral vector N2A+tRNA_i_^met^ and introduced into human U-87MG.EGFRvIII glioblastoma cells which overexpress EGFRvIII. N2A+tRNA_i_^met^ was chosen for this purpose because of the extreme transcription efficacy of the inserted DNA sequence due to the approximately 100-fold abundance of pol III-transcribed tRNA molecules as compared to polyadenylated RNA on a molar basis, and the placement of the foreign gene outside of the retroviral transcriptional unit, thereby eliminating or at least reducing the negative effects which may be exerted by this viral unit on the activity of the internal promoter [17,18]. In addition, the chimeric tRNA-ribozyme design [19] confers on the transcript relative resistance against intracellular degradation which is of particular relevance since the process of substrate binding, cleavage, and product release can be typically done repeatedly by a single ribozyme molecule, thereby allowing cleavage of multiple substrate molecules [11,20]. Moreover, the applicability of pol III transcription termination sequences assures that non-ribozyme sequences which could interfere with the ribozyme’s secondary and tertiary structure are not added to its 3’-end [21,22]. Notably, a general advantage that is provided by the usage of a retroviral vector in the brain is based on the fact that retroviral vectors only integrate and express their genes in proliferating cells (*e.g.*, tumors). Cancer cells represent the predominant mitotic cell type in the brain, thus selective transduction of tumor cells is maximized whereas transduction of the non-dividing normal brain cells is minimal or absent [23].

In the study of Halatsch *et al.*, both transduced (including control infections) and non-transduced U-87MG.EGFRvIII cells were resistant against neomycin, and therefore, drug selection was not employed to eradicate non-transduced cells which is in analogy to a potential clinical situation. Thus, the data obtained on proliferation, clonogenicity, and *EGFR* mRNA expression did not reflect properties of a purely transduced cell population, but of presumably heterogenous populations consisting of transduced as well as a small fraction of non-transduced cells. The experimental conditions under which these mixed populations were generated included a virus:cell ratio of 40:1. Despite the heterogeneity, provirus integration and expression levels were similar among ribozyme-, disabled ribozyme- and mock-transduced cells; therefore, the observed differences in biological activity were unlikely secondary to diffences in provirus integration and expression. The catalytically disabled control ribozyme's lack of biological activity suggests that the inhibition of proliferation and anchorage-independent growth exerted by the original ribozyme on U-87MG.EGFRvIII cells was due to ribozyme-specific catalytic cleavage rather than due to an antisense effect.

A more than 90% reduction of *EGFRvIII* mRNA levels in U-87MG.EGFRvIII was found subsequent to ribozyme transfer. In addition, transduction of U-87MG.EGFRvIII with anti-EGFRvIII-sCYMV1 under relative serum starvation conditions amounted to 69% inhibition of EGFRvIII-mediated proliferation advantage. Yet, clonogenicity in soft agar or anchorage-independent growth (under identical relative serum starvation conditions) which serves as the best *in vitro* correlate to tumorigenicity was profoundly (>95%) suppressed by transfer of anti-*EGFRvIII*-sCYMV1. 

Taken together, it appears that in these test systems EGFRvIII affects the interaction of glioblastoma cells with their environment rather than cell proliferation directly and that these EGFRvIII-mediated effects may be specifically and efficiently intercepted by a targeted ribozyme, the growth-suppressing action of which is intensified in a three-dimensional environment. Noteworthy, overexpression of the truncated EGFR in U-87MG.EGFRvIII is comparable to levels seen in primary brain tumors [6].

In another study, Yamazaki *et al.* have demonstrated specific hammerhead ribozyme-mediated cleavage of a synthetic *EGFRvIII* RNA substrate in a cell-free system [10]. Efficient catalytic activity of this ribozyme was observed at Mg^2+^ concentrations as low as 1 mM. Although the cleavage reaction has an absolute requirement for Mg^2+^ and the intracellular Mg^2+^ concentration appears suboptimal for ribozyme activity, other divalent catalysts, e.g., Ca^2+^, also promote formation of a catalytically proficient ribozyme-substrate complex *in vivo* [15]. Subsequently, this group constructed a hammerhead ribozyme expression plasmid which was introduced into ERM5-1 cells (*i.e.*, murine NIH/3T3 cells transfected to express *EGFRvIII* cDNA) *in vitro* using calcium phosphate coprecipitation. The transfected ribozyme suppressed expression of *EGFRvIII* mRNA and reduced the growth of tumors formed by transduced ERM5-1 cells in nude mice. The tumors formed by transduced ERM5-1 cells were at maximum one tenth the size of the tumors formed by the original ERM5-1 cells. The authors also showed that the mitotic activity of ribozyme-expressing ERM5-1 cells, as measured by bromodeoxyuridine incorporation, was decreased *in vivo*.

EGFRvIII is known to be also expressed by other human cancers, such as primary breast carcinoma. Luo *et al.* used an EGFRvIII-targeted ribozyme-based approach with breast cancer cell lines [24]. A synthetic hammerhead ribozyme was created and shown to be able to cleave a synthetic substrate in a cell-free system. Moreover, MCF-7/EGFRvIII cells and MDA435/LCC6 cells transfected with the ribozyme showed significant downregulation of both *EGFRvIII* mRNA and protein expression. In addition, tumors grown in mice after orthotopic implantation of ribozyme-transfected MDA435/LCC6 cells showed significantly reduced size as well as inhibited expression of EGFRvIII protein compared to tumors arising from untransfected or mock-transfected cells.

In summary, the findings of these studies indicate that anti-*EGFRvIII* ribozymes are capable of specifically inhibiting the expression of EGFRvIII and reversing the EGFRvIII-associated malignant phenotype of glioblastoma cells. Thus, this strategy may constitute a promising gene therapeutic approach for a molecularly defined subgroup of glioblastoma multiforme and other EGFRvIII-expressing human cancers.

## 4. Conclusions

EGFRvIII is the most common mutant form of HER1/EGFR. In various human malignant tumors, including high-grade glioma, expression of EGFRvIII is present in large subsets of neoplasms, and its presence was shown to be related to increased tumorigenicity. Absence of EGFRvIII expression in non-cancerous cells makes this receptor an interesting target for therapeutic strategies. A variety of different therapeutic approaches targeted at EGFRvIII have been developed. Ribozyme-mediated targeting of EGFRvIII expression as an anticancer strategy appears attractive due to the unsurpassed specificity of the approach. However, pharmacokinetic factors such as low transduction efficiency may limit the success of this approach as indicated by the results of the herpes simplex virus-thymidine kinase gene/ganciclovir (HSV-TK/GCV) trial on glioblastoma multiforme [23]. The anti-*EGFRvIII* ribozyme approach seems to cause rather a cytostatic than a cytocidal effect as opposed to the HSV-TK/GCV strategy, and a therapeutic benefit may be rather derived from increased susceptibility of targeted cancer cells towards conventional and new therapeutic modalities and agents.

Unfortunately, so far clinical data from studies evaluating the effects of other EGFRvIII-targeted agents have been disappointing. In the first randomized, controlled phase II trial of erlotinib in recurrent glioblastoma multiforme, only 11.4% of the study patients were free of progression after six months, compared to 24.1% of the control patients treated with temozolomide and BCNU [25]. Moreover, there was no significant difference in overall survival between the two treatment groups (7.7 months for the erlotinib group *versus* 7.3 months for the temozolomide/BCNU group). While one might speculate that insufficient drug delivery may underly the missing clinical benefit, knocking out only one oncogene may just not be the appropriate strategy to successfully conquer glioblastoma multiforme. After quite some lessons learned, hopes are now set on multi-targeting approaches to enhance treatment outcomes in this disease.
